# Decreased cerebral blood flow and improved cognitive function in patients with end-stage renal disease after peritoneal dialysis: An arterial spin-labelling study

**DOI:** 10.1007/s00330-018-5675-9

**Published:** 2018-08-13

**Authors:** Ben-Chung Cheng, Po-Cheng Chen, Pei-Chin Chen, Cheng-Hsien Lu, Yu-Chi Huang, Kun-Hsien Chou, Shau-Hsuan Li, An-Ni Lin, Wei-Che Lin

**Affiliations:** 1 Department of Nephrology, Kaohsiung Chang Gung Memorial Hospital and Chang Gung University College of Medicine, 123 Ta-Pei Road, Niao-Sung, Kaohsiung, Taiwan; 2 Department of Biological Science, National Sun Yat-Sen University, Kaohsiung, Taiwan; 3 Department of Physical Medicine and Rehabilitation, Kaohsiung Chang Gung Memorial Hospital and Chang Gung University College of Medicine, 123 Ta-Pei Road, Niao-Sung, Kaohsiung, Taiwan; 4 Department of Diagnostic Radiology, Kaohsiung Chang Gung Memorial Hospital and Chang Gung University College of Medicine, 123 Ta-Pei Road, Niao-Sung, Kaohsiung, Taiwan; 5 Department of Neurology, Kaohsiung Chang Gung Memorial Hospital and Chang Gung University College of Medicine, 123 Ta-Pei Road, Niao-Sung, Kaohsiung, Taiwan; 6 Brain Research Center, National Yang-Ming University, Taipei, Taiwan; 7 Institute of Neuroscience, National Yang-Ming University, Taipei, Taiwan; 8 Department of Internal Medicine, Kaohsiung Chang Gung Memorial Hospital and Chang Gung University College of Medicine, 123 Ta-Pei Road, Niao-Sung, Kaohsiung, Taiwan

**Keywords:** Peritoneal dialysis, Cognitive impairment, Arteries, Spin labelling, Cerebral blood flow

## Abstract

**Objectives:**

The aim of this study was to evaluate the relationship between cognitive impairment and brain perfusion using arterial spin labelling (ASL) in end-stage renal disease (ESRD) patients undergoing PD.

**Methods:**

ESRD patients undergoing PD were recruited. Laboratory screening, neuropsychological tests and ASL magnetic resonance imaging (MRI) were conducted prior to and after 6 months of PD. Age- and sex-matched normal subjects without ESRD served as the control group. Comparisons of regional CBF between ESRD patients before or after undergoing PD and normal controls were performed. Correlations between biochemical, neuropsychological and CBF data were also conducted to evaluate the relationships.

**Results:**

ESRD patients showed poor performance in many of the neuropsychological tests; PD improved cognition in some domains. Pre-PD patients had higher mean CBF than post-PD patients and normal controls, but no significant difference was found between the normal controls and post-PD patients. Negative correlations were observed pre-PD (regional CBF in left hippocampus vs. perseverative responses, r = -0.662, *p* = 0.014), post-PD (mean CBF vs. haemoglobin level, r = -0.766, *p* = 0.002), and before and after PD (change in CBF in the left putamen vs. change in haematocrit percentage, r = -0.808, *p* = 0.001).

**Conclusion:**

Before PD, ESRD patients had increased cerebral perfusion that was related to poorer executive function, especially in the left hippocampus. Post-PD patients performed better in some cognitive test domains than pre-PD patients. The degree of anaemia, i.e., haemoglobin level or haematocrit percentage, might predict cognitive impairment in PD patients.

**Key Points:**

*• In this study, ESRD patients before PD had cerebral hyperperfusion that was related to poorer executive function.*

*• Post-PD patients performed better in some cognitive test domains than pre-PD patients did.*

*• The degree of anaemia might predict cognitive impairment in PD patients.*

**Electronic supplementary material:**

The online version of this article (10.1007/s00330-018-5675-9) contains supplementary material, which is available to authorized users.

## Introduction

Chronic kidney disease (CKD), defined as abnormalities in kidney structure or function persisting for over 3 months, is a growing public health problem [1]. CKD staging is based on glomerular filtration rate (GFR) [1]. CKD stage 5, which requires dialysis or renal transplantation, is known as end-stage renal disease (ESRD). According to one cohort study from Taiwan, the prevalence of CKD is approximately 12%, of which ESRD accounts for 0.6% [2]. There are many complications associated with CKD, and cognitive impairment is commonly seen in ESRD patients [3, 4]. The prevalence of cognitive impairment in patients with ESRD ranges from 16% to 38% [5]. Additionally, patients undergoing haemodialysis (HD) or peritoneal dialysis (PD) tend to have a 2.5 times or higher probability of having moderate to severe cognitive impairment [4]. Due to high rates of dialysis, cognitive impairment in ESRD patients is gaining more attention in Taiwan.

The aetiology and pathophysiology of cognitive impairment in ESRD patients is still not clearly understood. There are many potential risk factors that can result in cognitive impairment in ESRD patients, such as ageing, diabetes mellitus, hypertension, hypercholesterolaemia, uraemia, anaemia, hyperhomocysteinaemia and cystatin-C [6–10]. In the 3C study, global cognitive decline with vascular dementia was associated with faster estimated GFR (eGFR) decline [11], which suggested a potential vascular aetiology for cognitive impairment in ESRD patients. Some researchers have found an association between poor renal function and cognitive impairment in the so-called kidney-brain axis in recent years [3, 12, 13]. In terms of anatomy and vasoregulation, both kidneys and the brain have circulation systems with low vascular resistance, exposing them to high-volume blood flow, and thus making them vulnerable to vascular damage [14]. This physiological phenomenon can induce cerebral hyperperfusion and cause cerebrovascular diseases, especially in Asian individuals [15–17].

Cerebral blood flow (CBF) can be detected by arterial spin labelling (ASL), a non-invasive magnetic resonance imaging (MRI) perfusion tool that utilises arterial blood water as a freely diffusible endogenous tracer to quantify the CBF per unit of tissue mass [18]. This technique has been applied to many diseases, such as stroke [19], brain tumours [20] and epilepsy [21]. In recent years, advances in both the pulse sequence design and high-field MRI technology improve the signal-to-noise ratio (SNR) and therefore increase utilisation in clinical neuroimaging. ASL MRI has also been used to investigate the changes in CBF in HD patients [22–24]. PD differs from HD in terms of its haemodynamic characteristics in the regulation of body fluid [25]. Therefore, regulation of CBF should be different in patients undergoing PD than in those undergoing HD. One recently published article discussed the CBF measured by ASL MRI in patients undergoing PD [26]. In this study [26], PD patients had widespread regional CBF decline compared with non-dialysis ESRD patients. However, the study was not designed to measure the changes in CBF that occurred as a result of PD.

The aim of this study was to investigate the effect of PD on CBF and cognitive function in ESRD patients. ASL MRI was used to evaluate cerebral perfusion in PD patients before and after PD. Additionally, significant changes in CBF in specific regions of interest (ROIs) were analysed, and the possible correlations between biochemical parameters and cognitive performance were assessed.

## Materials and methods

### Participants

This prospective study recruited consecutive patients with ESRD who underwent PD. The inclusion criteria were (1) patients diagnosed with ESRD, with disease duration longer than 3 months; and (2) patients undergoing PD for longer than 3 months. The exclusion criteria were: (1) age less than 18 years; (2) presence of brain lesions, such as stroke, tumours or abscesses, as determined based on the patient’s medical history or conventional MRI; (3) history of traumatic brain injury; (4) major neuropsychiatric disorders; or (5) history of drug or alcohol abuse.

All outcome variables, including laboratory screening, neuropsychological test scores and MRI scans, were measured before commencement of PD and on one day after 6 months of PD. Fifteen patients with ESRD (eight males and seven females; mean age 57.07 ± 9.18 years) were included. Another 18 age- and sex-matched normal control subjects (ten males and eight females; mean age 57.56 ± 7.56 years), who had no relevant medical history or neurological diseases, served as the control group, and were recruited through advertisement within the hospital. The individuals in the control group only underwent neuropsychological tests and MRI.

Chang Gung Memorial Hospital’s Institutional Review Committee on Human Research approved the project. All participants were informed that the study was designed to evaluate the change in cognition before and after undergoing 6 months of PD. All subjects provided written informed consent prior to participation.

### Laboratory examinations

Blood biochemical tests, including haemoglobin, haematocrit, serum urea, serum creatinine, estimated glomerular filtration rate, calcium, phosphorus and albumin levels, were conducted in all patients with ESRD before and after undergoing PD for 6 months. The control group did not undergo any blood biochemical tests.

### Neuropsychological tests

A battery of neuropsychological tests focusing on attention, executive function, memory, speech and language function, and visuospatial function were performed in all subjects within 1 h before or after having MRI. Different neuropsychological domains were measured by subtests from the Cognitive Ability Screening Instrument (CASI) [27], Wechsler Adult Intelligence Scale-third edition (WAIS-III) [28] and Wisconsin Card Sorting Test (WCST-64, Computer Version Scoring Program) [29]. The CASI and the mini-mental state examination (MMSE) total scores were used for evaluation of overall cognitive function.

### Evaluation of cerebral perfusion

#### MRI data acquisition

MRI data acquisition and processing were performed as previously reported [30, 31], and the procedures are summarised in the Online Supplementary Material.

#### Image data processing

Imaging data were preprocessed using FSL v5.0 (Functional Magnetic Resonance Imaging of the Brain Software Library; http://www.fmrib.ox.au.uk/fsl) and SPM8 (Statistical Parametric Mapping, Wellcome Department of Imaging Neuroscience, London, UK; available online at http://www.fil.ion.ucl.ac.uk/spm) implemented in Matlab 7.3 (MathWorks, Natick, MA, USA). The processing procedures are documented in the Online Supplementary Material.

### Statistical analysis

#### Analysis of demographic data

Numerical demographic data, including age, body mass index (BMI), education level, neuropsychological test scores and global brain measurements (total intracranial volume (TIV) and mean CBF) were reported as mean ± standard deviation and compared between the pre-PD (ESRD patients before beginning PD) and normal control groups using Mann-Whitney U tests. The same comparisons were also conducted between the post-PD (ESRD patients after undergoing PD for 6 months) and normal control groups. For the paired pre-PD and post-PD groups, numerical variables, including the biochemical parameters, neuropsychological test scores and the global tissue volumes of the brain, were reported as mean ± standard deviation and compared using Wilcoxon signed-rank tests. Statistical significance was set at *p* < 0.05. Statistical analysis was performed using the Statistical Package for the Social Sciences (SPSS) software package (version 17; SPSS Inc., Chicago, IL, USA).

#### Analysis of regional CBF differences between groups

The differences in CBF maps were analysed between the following groups: (1) pre-PD versus normal control; (2) post-PD versus normal control; and (3) pre-PD versus post-PD. The comparing methods are described in the Online Supplementary Material.

#### Analysis of regions of interest

Based on the whole-brain voxel-wise comparisons, analysis of ROI was conducted to determine the mean CBF value of each significantly different area between the groups. The MarsBaR toolbox (http://marsbar.sourceforge.net/download.html) was used to extract the ROI masks. The ROI was defined as an area with significant difference in CBF.

#### Correlations between biochemical, neuropsychological and CBF data

Partial correlation was performed to correlate biochemical parameters, neuropsychological test scores, mean CBF and regional CBF by adjusting for age and sex. In patients with ESRD, differences in biochemical parameters before and after undergoing PD were also used to correlate mean CBF and regional CBF. The statistical significance was set at *p* < 0.05, with Bonferroni correction for multiple comparisons.

## Results

### Demographic characteristics of the subjects

The demographic characteristics of the 15 ESRD patients and 18 normal controls are presented in Table 1. There were no significant differences in age, gender, education level or BMI between the pre-PD and normal control groups. With regard to the biochemical parameters, there were also no significant differences between the pre-PD and post-PD groups. The pre-PD group had significantly lower scores on the neuropsychological tests than the normal control group in the aspects of attention (mental manipulation, *p* = 0.033), executive function (digit symbol coding, *p* = 0.001), memory function (short-term memory, *p* = 0.040), speech and language function (comprehension, *p* = 0.006; similarities, *p* = 0.016), and visuospatial function (picture completion, *p* = 0.001; block design, *p* = 0.007). The post-PD group also had poorer cognitive function than the normal control group in many aspects, such as attention (mental manipulation, *p* = 0.036), executive function (digit symbol coding, *p* = 0.005), and visuospatial function (picture completion, *p* = 0.008; block design, *p* = 0.020). When it came to overall cognitive function, both the pre-PD and post-PD group had lower CASI and MMSE scores than the normal control group (*p* = 0.015 and *p* = 0.022 for pre-PD vs. normal control; *p* = 0.048 and *p* = 0.048 for post-PD vs. normal control, respectively). Comparisons of neuropsychological test scores between the paired pre-PD and post-PD groups showed significantly higher scores in the post-PD group for executive function (digit symbol coding, *p* = 0.032; number of categories completed, *p* = 0.039), memory function (short-term memory, *p* = 0.026), speech and language function (comprehension, *p* = 0.040), and visuospatial function (picture completion, *p* = 0.048) than in the pre-PD group. However, there were no significant differences in CASI or MMSE scores between the pre-PD and post-PD groups. Analysis of the global brain measurements found both the pre-PD and post-PD groups to have significantly higher mean CBF than the normal controls (*p* < 0.001 and *p* = 0.036, respectively). However, no significant differences were noted in TIV among the pre-PD, post-PD and normal control groups.

**Table 1:** Demographics, biochemical parameters and neuropsychological tests of ESRD patients before and after undergoing PD and normal controls

	Normal control	ESRD patients		*p* value		
		Pre-PD	Post-PD	NC vs. Pre-PD^a^	NC vs. Post-PD^b^	Pre-PD vs. Post-PD^c^
No. of subjects	18	15	15			
Age (y)	57.56 ± 7.56	57.07 ± 9.18	57.07 ± 9.18	0.789	-	-
Gender (male/female)	10/8	8/7	8/7	>0.999	-	-
Education (years)	11.89 ± 5.87	9.13 ± 3.18	9.13 ± 3.18	0.114	-	-
BMI (kg/m2)		26.29 ± 5.80	26.77 ± 10.91	-	-	0.363
Biochemical parameters						
Haemoglobin (g/dl)	-	9.11 ± 1.48	10.09 ± 1.64	-	-	0.053
Hct (%)	-	27.45 ± 4.46	29.68 ± 5.38	-	-	0.125
BUN (mg/dl)	-	76.47 ± 26.41	69.53 ± 24.32	-	-	0.222
Creatinine (mg/dl)	-	11.02 ± 4.72	10.64 ± 3.57	-	-	0.865
EGFR (ml/min/1.73 m2)	-	4.73 ± 1.98	4.80 ± 2.11	-	-	0.944
Ca (mg/dl)	-	9.00 ± 1.23	8.78 ± 0.76	-	-	0.925
P (mg/dl)	-	4.97 ± 1.41	5.03 ± 1.59	-	-	0.609
Albumin (g/dl)	-	3.43 ± 0.48	3.49 ± 0.42	-	-	0.198
Neuropsychological tests						
Attention						
Mental manipulation (CASI)	9.28 ± 1.36	7.80 ± 2.14	8.00 ± 1.69	0.033*	0.036*	0.751
Attention (CASI)	7.22 ± 1.06	6.87 ± 0.99	7.07 ± 0.88	0.290	0.464	0.490
Orientation (CASI)	17.28 ± 1.41	17.07 ± 2.37	17.00 ± 1.65	0.957	0.605	0.660
Executive function						
Digit symbol coding (WAIS)	9.94 ± 3.65	5.64 ± 2.65	6.53 ± 2.56	0.001**	0.005**	0.032*
Abstract thinking (CASI)	9.78 ± 1.96	9.47 ± 1.77	9.00 ± 1.96	0.656	0.274	0.412
Total number correct (WCST)	35.59 ± 12.03	34.53 ± 12.22	39.33 ± 10.06	0.823	0.274	0.053
Total number error (WCST)	28.41 ± 12.03	23.33 ± 16.86	24.67 ± 10.06	0.823	0.390	0.865
Perseverative responses (WCST)	13.06 ± 6.87	17.47 ± 8.48	13.07 ± 5.40	0.100	0.789	0.099
Perseverative errors (WCST)	11.89 ± 5.73	14.80 ± 6.03	11.60 ± 4.37	0.145	0.929	0.060
Non-perseverative errors (WCST)	15.44 ± 10.10	14.67 ± 9.69	13.07 ± 9.13	0.873	0.464	0.570
Conceptual level responses (WCST)	0.44 ± 0.27	0.41 ± 0.26	0.48 ± 0.21	0.817	0.630	0.158
Number of categories completed (WCST)	2.22 ± 1.67	1.53 ± 1.06	2.13 ± 1.25	0.343	0.957	0.039*
Memory function						
Long-term memory (CASI)	9.78 ± 0.94	9.33 ± 0.98	9.20 ± 1.82	0.215	0.486	>0.999
Short-term memory (CASI)	9.94 ± 1.82	8.01 ± 2.81	9.41 ± 1.85	0.040*	0.381	0.026*
Speech and language function						
Language (CASI)	9.50 ± 1.03	8.96 ± 0.99	9.35 ± 0.80	0.057	0.178	0.194
Semantic verbal fluencies (CASI)	7.83 ± 2.31	6.40 ± 2.23	6.93 ± 2.37	0.086	0.290	0.429
Comprehension (WAIS)	10.35 ± 3.32	6.64 ± 3.22	7.53 ± 3.58	0.006**	0.067	0.040*
Similarities (WAIS)	10.17 ± 2.94	7.80 ± 1.93	8.53 ± 2.13	0.016*	0.100	0.184
Visuospatial function						
Picture completion (WAIS)	10.44 ± 4.73	6.20 ± 2.14	7.20 ± 1.47	0.001**	0.008**	0.048*
Block design (WAIS)	10.11 ± 3.60	6.73 ± 2.66	7.13 ± 2.85	0.007**	0.020*	0.281
Drawing (CASI)	9.72 ± 0.67	9.87 ± 0.35	9.80 ± 0.56	0.817	0.873	0.705
CASI total score	90.33 ± 8.24	83.77 ± 7.80	85.69 ± 8.47	0.015*	0.048*	0.293
MMSE total score	27.22 ± 2.65	25.27 ± 2.60	25.87 ± 1.55	0.022*	0.048*	0.226
Global brain measurements						
TIV (ml)	1363.69 ± 138.70	1289.32 ± 117.24	1293.32 ± 120.87	0.117	0.166	0.256
Mean CBF (mL/100g/min)	40.81 ± 5.33	54.65 ± 11.00	51.21 ± 16.42	<0.001***	0.036*	0.394

Numerical data are presented as mean ± standard deviation, and categorical data are presented as numbers

^a, b^Mann-Whitney U test

^c^Wilcoxon signed-rank test

*ESRD* end-stage renal disease, *PD* peritoneal dialysis, *BMI* body mass index, *Hct* haematocrit, *BUN* blood urea nitrogen, *EGFR* estimated glomerular filtration rate, *Ca* calcium, *P* phosphorus, *CASI* Cognitive Ability Screening Instrument, *WAIS* Wechsler Adult Intelligence Scale, *WCST* Wisconsin Card Sorting Test, *MMSE* Mini-Mental State Examination, *TIV* total intracranial volume, *CBF* cerebral blood flow

* *p* < 0.05, ** *p* < 0.01, *** *p* < 0.001

### Brain perfusion differences between groups

#### Between the pre-PD and normal control groups

**Fig. 1:**
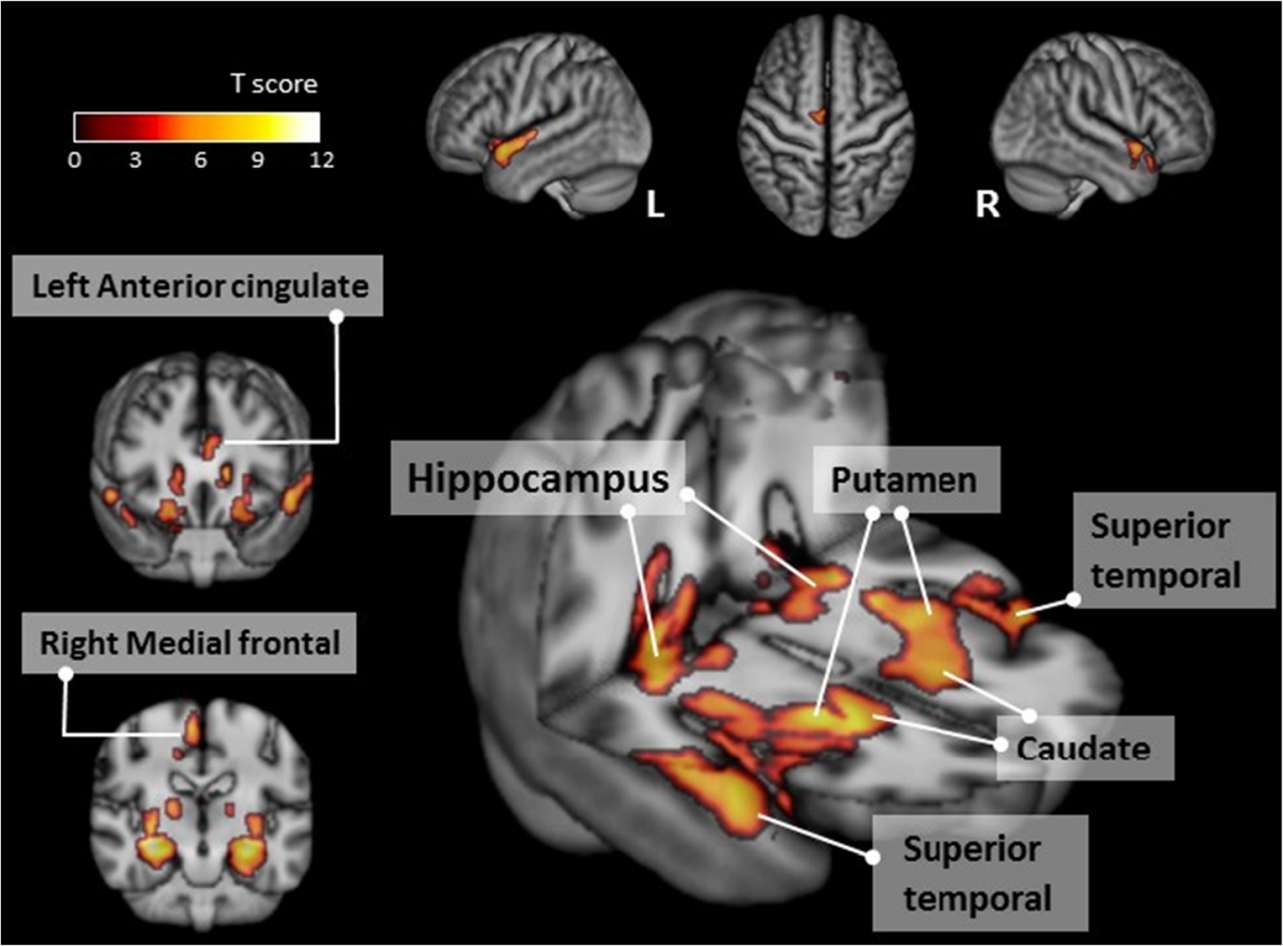
Regions with significantly higher cerebral blood flow in the pre-PD group compared to the normal control group (cluster level statistics, *p* < 0.05, family-wise error corrected). The colour bar indicates the T score

Voxel-wise analysis of the absolute CBF maps revealed a significantly higher regional CBF value of the bilateral limbic systems, bilateral temporal lobes and the right frontal lobe in the pre-PD group than in the normal control group (Table 2; Fig. 1).

**Table 2:** Comparisons of CBF in different brain regions between ESRD patients before or after undergoing PD and normal controls

MNI atlas coordinates			Voxel size	Region		T_max_
X	Y	Z		Anatomy	Brodmann area	
Pre-PD > NC (p_FWE_ < 0.05)						
32	-19	-14	6,721	Right hippocampus, caudate, putamen	-	12.23
-27	-16	-15	8,232	Left hippocampus, caudate, putamen	-	10.91
-52	9	-11	530	Left superior temporal gyrus	22	8.86
8	-12	64	582	Right medial frontal gyrus	6	8.6
56	9	-9	482	Right superior temporal gyrus	22	8.37
-4	36	12	401	Left anterior cingulate	24	7.23
Post-PD > NC (p_FWE_ < 0.05)						
-	-	-	-	-	-	-
Pre-PD > Post-PD (p_uncorrected_ < 0.001 with a cluster size > 20 voxels)						
-38	24	31	94	Left middle frontal gyrus	9	5.63
-34	56	-5	104	Left middle frontal gyrus	10	5.55
-48	-57	1	46	Left inferior temporal gyrus	19	5.27
-50	-42	42	40	Left inferior parietal lobule	40	5.25
-9	-79	-6	61	Left lingual gyrus	18	5.23
39	20	12	64	Right insula	13	5.1
-56	-9	33	39	Left precentral gyrus	4	5.03
-42	27	6	32	Left inferior frontal gyrus	13	4.91
-33	21	-24	43	Left inferior frontal gyrus	47	4.63
-27	-7	3	31	Left putamen	-	4.63
-57	-61	18	38	Left middle temporal gyrus	39	4.36

*CBF* cerebral blood flow, *ESRD* end-stage renal disease, *PD* peritoneal dialysis, *MNI* Montreal Neurological Institute, *NC* normal controls, *FWE* familywise error correction, *T*_*max*_ time to maximum concentration

#### Between the post-PD and normal control groups

No specific signals were detected (Table 2).

#### Between the pre-PD and post-PD groups

**Fig. 2:**
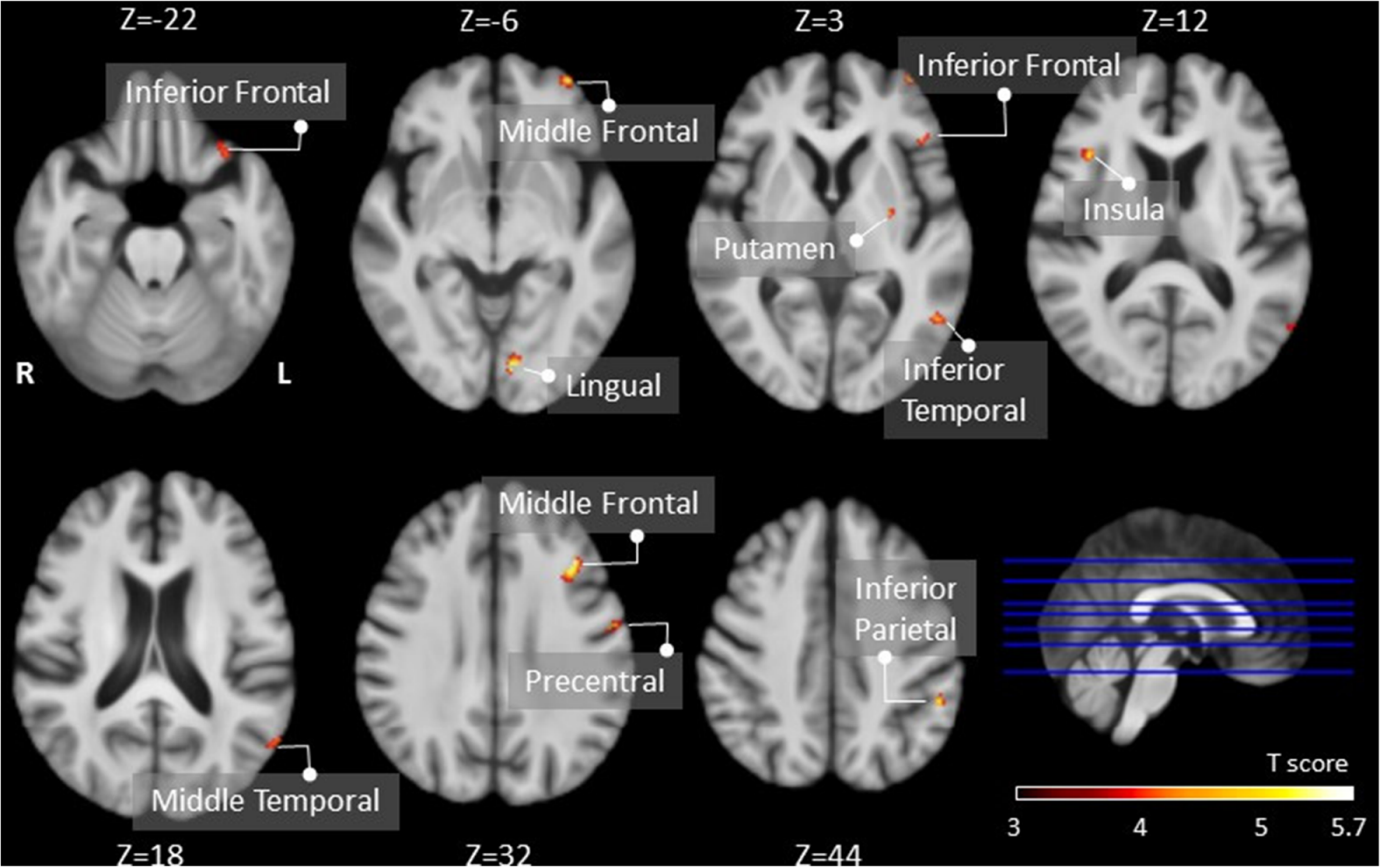
Regions with significantly decreased cerebral blood flow in the post-PD group compared to the pre-PD group (cluster level statistics, *p* < 0.001 with a cluster size > 20 voxels, uncorrected). The colour bar indicates the T score

The post-PD group showed significantly lower CBF in the left frontal, parietal and temporal lobes, the left putamen and the right insula than the normal control group (Table 2; Fig. 2).

### Correlations between biochemical, neuropsychological, and CBF in patients with ESRD

#### Pre-PD group

**Fig. 3:**
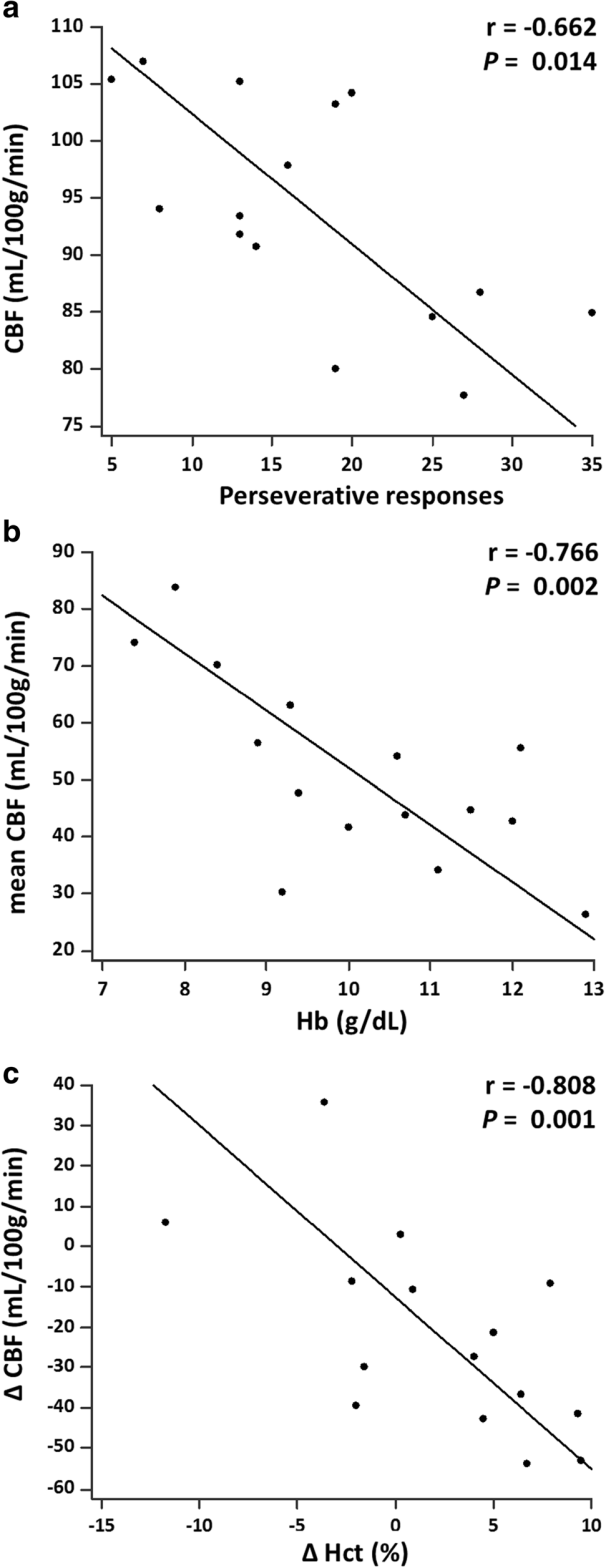
Partial correlations between (**a**) CBF at the left hippocampus and perseverative responses scores in the pre-PD group, (**b**) mean CBF and haemoglobin level in the post-PD group, and (**c**) change in regional CBF at the left putamen and change in haematocrit percentage between the pre-PD and post-PD groups

Regional CBF in the left hippocampus was negatively correlated with perseverative responses (one item of executive function in the WCST) (r = -0.662, *p* = 0.014) (Fig. 3a). No other significant correlation was found.

#### Post-PD group

Mean CBF was negatively correlated with the haemoglobin level (r = -0.766, *p* = 0.002) (Fig. 3b). No other significant correlation was found.

#### Pre-PD and post-PD groups

The change in CBF in the left putamen was negatively correlated with the change in haematocrit percentage (r = -0.808, *p* = 0.001) (Fig. 3c). No other significant correlation was found.

## Discussion

ESRD patients usually experience cognitive impairment, even after dialysis [3–5, 32]. In our study, disturbances in attention, executive function, memory function, speech and language function, and visuospatial function were noted in the ESRD patients. However, research on the effect of PD on cognitive function is rare. In one recent cross-sectional study [26], PD and non-dialysis patients were found to perform similarly on neuropsychological tests. However, the study did not include a paired before-and-after comparison of the effect of PD on cognitive function. In our study, the post-PD group scored higher on tests of executive function, memory function, speech and language function, and visuospatial function. All of these findings are supported by previous neuroimaging studies that used voxel-based morphometry [33, 34], resting-state functional MRI [35, 36], and diffusion-tensor imaging [37]. Our study used ASL MRI to study the effect of brain perfusion on cognitive impairment. Higher regional CBF of the bilateral limbic systems, bilateral temporal lobes and right frontal lobe in the pre-PD group was noted in comparison with the normal control group. The post-PD group also showed lower regional CBF in the left frontal, parietal and temporal lobes, left putamen, and right insula than the pre-PD group. These results indicate certain associations between the change in regional CBF and specific cognitive function, which supports the hypothesis of a vascular aetiology of cognitive impairment in ESRD patients [11]. ESRD patients also had higher mean CBF values than the normal controls; therefore, generalised vascular damage of the brain could be inferred.

In the pre-PD group, regional CBF in the left hippocampus was negatively correlated with perseverative responses, which was one test of executive function. Previous studies have reported a relationship between the hippocampus and executive function in other diseases [38, 39], and hippocampal volume loss could be related to executive dysfunction. We hypothesised that cerebral hyperperfusion in this region would lead to vascular damage, resulting in neuroplastic changes and reduced hippocampal volume. A recent pilot study also discovered hippocampal atrophy in HD patients [40]. Therefore, the hippocampus is thought to be a region responsible for cognitive impairment in ESRD patients. In the post-PD group, an inverse correlation was recognised between the global mean CBF and haemoglobin levels. Previous studies using computed tomography [41, 42], positron emission tomography [43] and phase contrast MRI [44] have reported similar findings. Our findings were consistent with a recently published study that also used ASL MRI [26]. Both brain tissue hypoxia and a reduction in cerebral vasodilatory capacity could contribute to this phenomenon, though the dominant factor remains unclear [45]. Anaemia could cause cerebral hyperperfusion, resulting in consequent cognitive dysfunction; increasing evidence supports the hypothesis that correction of anaemia in dialysis patients can improve cognitive function [9, 46–48].

In ESRD patients before and after PD, the change in CBF in the left putamen was negatively correlated with the change in haematocrit percentage. Haematocrit has been shown to play a key role in the cognitive function of dialysis patients [47]. Normalising haematocrit levels might decrease regional CBF, thus reducing vascular damage in the brain. In our study, a decrease in regional CBF occurred in the left putamen; however, this association has not been reported in the literature. The putamen is characterised by high neuronal density and rich vascularisation, and is supplied by the anterior and middle cerebral arteries, all of which make it vulnerable to vascular damage [49]. A growing body of evidence has found that the putamen is crucial not only for motor skills, but also for learning [50]. Therefore, we hypothesise that the correction of haematocrit levels improved cognitive function, particularly learning.

In our study, cerebral hyperperfusion was observed in ESRD patients and was related to cognitive impairment. Dysregulation of the cerebral vascular system and hypertension are possible pathophysiological mechanisms for cerebral hyperperfusion. One animal study described a correlation between an ischaemic cortex and impaired CBF autoregulation [51]. Because many ESRD patients have a risk of silent brain infarctions [52], these patients might have poor CBF autoregulation. In addition, the brain and kidneys share many physiological features, such as low vascular resistance, which makes them susceptible to vascular damage [14]. ESRD patients have chronic arterial hypertension due to several multifactorial aetiologies [53]. Long-standing hypertension leads to endothelial dysfunction and microangiopathy, resulting in eventual breakdown of the blood brain barrier (BBB). One animal model study showed that cerebral hyperperfusion occurred after breakdown of the BBB [54]. It is believed that extravasation of serum albumin after BBB breakdown activates the transforming growth factor beta-signalling pathway, and that this phenomenon can cause cerebral oedema and hyperperfusion. Non-traditional vascular risk factors, such as hyperhomocysteinaemia, hypercoagulable states, inflammation and oxidative stress, have been linked to cognitive impairment [8]. These factors might accelerate the progression of atherosclerosis and vascular endothelial dysfunction and thus exacerbate cognitive decline through cerebral hyperperfusion.

Because haemoglobin and haematocrit might affect the results of comparison between pre-PD and post-PD patients, we conducted further comparisons adjusted by haemoglobin and haematocrit (results not shown). After adjustment, some regions with a nonsignificant difference in CBF might be related to the principle of imaging in ASL MRI (ASL uses arterial water protons labelled by radiofrequency pulses, and lower haemoglobin or haematocrit level is related to higher water content and thus stronger signal intensity of ASL). However, the main results remained showing significantly lower CBF of left frontal lobe, left putamen and right insula in post-PD patients. Although level of haemoglobin and haematocrit might be related to the clinical cognitive performance in patients with CKD [44], further validation supported our hypothesis that ASL could effectively detect the effect of PD treatment on brain perfusion in particular brain region that potentially altered the cognitive functions.

There were some strengths to our study. First, it is the first study to conduct a comparison before and after PD in ESRD patients using ASL MRI. Although one previous study [26] compared PD patients to non-dialysis patients, the results might have been affected by selection bias. Second, we used more biochemical parameters than the previous study [26]. Third, a battery of neuropsychological tests was conducted to test all domains of cognition as precisely as possible. However, several limitations of our study should be recognised. First, the small sample size might limit the statistical power of presenting differences among groups. Second, the study design was longitudinal only for the case group. The effect of PD on cognitive function was not examined in ESRD patients who did not undergo dialysis for 6 months. However, in clinical practice, it is nearly impossible to find ESRD patients who do not undergo dialysis treatment for 6 months. Finally, the different histopathological types of nephropathy in ESRD patients might affect CBF differently, but we did not consider them as covariates. Future studies that compare the different nephropathies and their roles in cognitive impairment are necessary.

In conclusion, ESRD patients had higher CBF than normal controls before undergoing PD, which was related to cerebral hyperperfusion. Increased CBF in the left hippocampus was noted in these patients, and it was related to poorer executive function. After PD, ESRD patients had better cognition in some domains than the ESRD patients before PD and a significant difference in some regional CBF was noted. The degree of anaemia, i.e. the haemoglobin level or haematocrit percentage, might be predictive of cognitive impairment in PD patients. Future studies should focus on the relationship between different types of nephropathies and cognitive impairment.

## Electronic supplementary material

ESM 1 (DOCX 52 kb)

## Abbreviations

BBB: Blood brain barrier
BMI: Body mass index
CASI: Cognitive Ability Screening Instrument
CBF: Cerebral blood flow
CKD: Chronic kidney disease;
eGFR: Estimated GFR
ESRD: End-stage renal disease;
GFR: Glomerular filtration rate
GLM: General linear model
GM: Grey matter
HD: Haemodialysis
MMSE: Mini-mental state examination
MNI: Montreal Neurological Institute
PD: Peritoneal dialysis
PVE: Partial volume effect
TIV: Total intracranial volume
WAIS-III: Wechsler Adult Intelligence Scale-third edition
WCST-64: Wisconsin Card Sorting Test
WM: White matter
