# Supplementary material for: Decreased cerebral blood flow and improved cognitive function in patients with end-stage renal disease after peritoneal dialysis: An arterial spin-labelling study
Source: Eur Radiol. 2018 Aug 13;29(3):1415–24. doi: 10.1007/s00330-018-5675-9 (PMC6510858; doi:10.1007/s00330-018-5675-9)
Supplement: Supplementary file 1 — (DOCX 52 kb) [file 330_2018_5675_MOESM1_ESM.docx]

**Supplementary document**

*2.4.1 MRI data acquisition*

MRI data acquisition and processing were performed as previously reported [1; 2]. High resolution T1-weighted imaging (T1WI) was conducted on a 3.0 T scanner (GE Signa MRI, Milwaukee, WI, USA) with an eight-channel head coil. Perfusion MRI was conducted on a 1.5 T scanner (Discovery MR450, GE Healthcare, Milwaukee, WI, USA) with an eight-channel head coil. A pulsed continuous ASL technique was used to obtain the images [3]. The imaging parameters were: TR = 4,548 ms, post-label delay = 1,525 ms, TE = 10.5 ms, matrix size = 128 × 128, number of excitations = 3, number of slices = 38, slice thickness = 4.0 mm (with whole-brain coverage), and total acquisition time = 4 min. Two images were acquired through an ASL perfusion imaging sequence: one taken shortly after the inflowing arterial spins were inverted (labeled image) and one taken without inverting the arterial spins (unlabeled image). Subtraction of the labeled image from the unlabeled image produced an ASL perfusion weighted image, which was converted to a quantitative image that reflected CBF. For each subject, a CBF map was calculated using a scanner console with FuncTool 3DASL (GE Healthcare) within 1 min, and CBF was reported in units of ml/100 g/min.

*2.4.2 Image data processing*

Imaging data were preprocessed using FSL v5.0 (Functional Magnetic Resonance Imaging of the Brain Software Library; http://www.fmrib.ox.au.uk/fsl) and SPM8 (Statistical Parametric Mapping, Wellcome Department of Imaging Neuroscience, London, UK; available online at http://www.fil.ion.ucl.ac.uk/spm) implemented in Matlab 7.3 (MathWorks, Natick, MA, USA). An experienced neuroradiologist meticulously checked all T1WI and ASL images from each subject to ensure that no scanner artifacts, motion problems, or gross anatomic abnormalities existed in the images. In order to further ensure the accuracy of cross-modality image registration, the unlabeled ASL images and corresponding T1WI of each subject were skull-stripped using the Brain Extraction Tool v2.1 (BET and BET2; part of the FSL software) to remove non-brain tissues and background noise from the images [4]. For each subject, the brain mask generated from the unlabeled ASL images was also applied to the CBF map. A boundary-based registration algorithm implemented in FMRIB's Linear Image Registration Tool (FLIRT; part of the FSL software) was used to obtain accurate and robust ASL and T1WI alignment [5]. We used the high resolution T1WI as the reference image of the extracted surfaces to separate the different tissue types. The unlabeled ASL image was then aligned to the reference by maximizing the intensity gradient across tissue boundaries. The CBF maps were subsequently registered into the T1WI space using a transformation.

To generate group-specific templates for all the subjects based on their segmented gray matter (GM) and white matter (WM) probability maps, the DARTEL (Diffeomorphic Anatomical Registration Through Exponentiated Lie Algebra) [6] toolbox from SPM8 was utilized. Each group-specific template was registered into the Montreal Neurological Institute (MNI) standard space through affine transformation, and each individual subject's T1WI was mapped into the MNI space. CBF maps in the T1WI space were normalized to the group-speciﬁc template, mapped into the MNI space, and re-sliced to an isotropic voxel size of 1.5 mm with these two transformations. To avoid a partial volume effect (PVE), CBF maps were corrected for volume atrophy according to the proportion of GM and WM in each voxel acquired from the segmented T1WI [7]. The value of CBF was set at a threshold between a lower limit of zero and an upper limit of two standard deviations above the mean perfusion value for each subject [8] to eliminate outliers in the perfusion image resulting from large blood vessels or image processing computations. Finally, we used a 6-mm full-width at half-maximum Gaussian kernel to spatially smooth the CBF maps after PVE correction for voxel-wise comparisons.

*2.5.2 Analysis of regional CBF differences between groups*

The differences in CBF maps were analyzed between the following groups: (1) pre-PD vs. normal control; (2) post-PD vs. normal control; and (3) pre-PD vs. post-PD. PVE-corrected smoothed CBF maps were analyzed with SPM8, and utilized the framework of a general linear model (GLM) to identify CBF differences between the groups. For comparisons of pre-PD vs. normal control and post-PD vs. normal control, a voxel-wise analysis of covariance (ANCOVA) design was used with age, sex, and TIV as covariates to remove possible interaction effects. For comparisons of the pre-PD vs. post-PD groups, paired-t tests were used. The GM mask generated from the group-specific template, including voxels with a GM probability value < 0.2 (within a range of 0 to 1) was excluded to prevent possible PVE between boundaries of different tissue types. The coordinates of the voxels of each significant cluster were transformed from MNI coordinates into Talairach coordinates using the GingleALE toolbox (The BrainMap Development Team; http://brainmap.org/ale/index.html). The Talairach and Tournoux atlas (http://www.talairach.org/index. html) was used to identify the anatomical structures of the Talairach coordinates representing significant clusters.

**References**

1 Chou KH, Lin WC, Lee PL et al (2015) Structural covariance networks of striatum subdivision in patients with Parkinson's disease. Hum Brain Mapp 36:1567-1584

2 Lin WC, Chen PC, Huang CC et al (2017) Autonomic Function Impairment and Brain Perfusion Deficit in Parkinson's Disease. Front Neurol 8:246

3 Lin WC, Chen PC, Huang YC et al (2016) Dopaminergic Therapy Modulates Cortical Perfusion in Parkinson Disease With and Without Dementia According to Arterial Spin Labeled Perfusion Magnetic Resonance Imaging. Medicine (Baltimore) 95:e2206

4 Smith SM (2002) Fast robust automated brain extraction. Hum Brain Mapp 17:143-155

5 Greve DN, Fischl B (2009) Accurate and robust brain image alignment using boundary-based registration. Neuroimage 48:63-72

6 Ashburner J (2007) A fast diffeomorphic image registration algorithm. Neuroimage 38:95-113

7 Johnson NA, Jahng GH, Weiner MW et al (2005) Pattern of cerebral hypoperfusion in Alzheimer disease and mild cognitive impairment measured with arterial spin-labeling MR imaging: initial experience. Radiology 234:851-859

8 Wang Z, Aguirre GK, Rao H et al (2008) Empirical optimization of ASL data analysis using an ASL data processing toolbox: ASLtbx. Magn Reson Imaging 26:261-269
